# Public preferences for vaccination and antiviral medicines under different pandemic flu outbreak scenarios

**DOI:** 10.1186/s12889-015-1541-8

**Published:** 2015-02-27

**Authors:** Helena Rubinstein, Afrodita Marcu, Lucy Yardley, Susan Michie

**Affiliations:** Health Psychology Research Group, University College London, London, WC1E 7HB UK; School of Psychology, University of Southampton, Southampton, SO17 1BJ UK

**Keywords:** Pandemic influenza, Vaccination, Antiviral medicines, Behaviour

## Abstract

**Background:**

During the 2009-2010 A(H1N1) pandemic, many people did not seek care quickly enough, failed to take a full course of antivirals despite being authorised to receive them, and were not vaccinated. Understanding facilitators and barriers to the uptake of vaccination and antiviral medicines will help inform campaigns in future pandemic influenza outbreaks. Increasing uptake of vaccines and antiviral medicines may need to address a range of drivers of behaviour. The aim was to identify facilitators of and barriers to being vaccinated and taking antiviral medicines in uncertain and severe pandemic influenza scenarios using a theoretical model of behaviour change, COM-B.

**Methods:**

Focus groups and interviews with 71 members of the public in England who varied in their at-risk status. Participants responded to uncertain and severe scenarios, and to messages giving advice on vaccination and antiviral medicines. Data were thematically analysed using the theoretical framework provided by the COM-B model.

**Results:**

Influences on uptake of vaccines and antiviral medicines - capabilities, motivations and opportunities - are part of an inter-related behavioural system and different components influenced each other. An identity of being healthy and immune from infection was invoked to explain feelings of invulnerability and hence a reduced need to be vaccinated, especially during an uncertain scenario. The identity of being a ‘healthy person’ also included beliefs about avoiding medicine and allowing the body to fight disease ‘naturally’. This was given as a reason for using alternative precautionary behaviours to vaccination. This identity could be held by those not at-risk and by those who were clinically at-risk.

**Conclusions:**

Promoters and barriers to being vaccinated and taking antiviral medicines are multi-dimensional and communications to promote uptake are likely to be most effective if they address several components of behaviour. The benefit of using the COM-B model is that it is at the core of an approach that can identify effective strategies for behaviour change and communications for the future. Identity beliefs were salient for decisions about vaccination. Communications should confront identity beliefs about being a ‘healthy person’ who is immune from infection by addressing how vaccination can boost wellbeing and immunity.

**Electronic supplementary material:**

The online version of this article (doi:10.1186/s12889-015-1541-8) contains supplementary material, which is available to authorized users.

## Background

The 2009 A/(H1N1) influenza pandemic was less markedly severe than previous strains such as the H3N2 virus in 1968 [1]. The groups that were most at-risk from infection were those aged below 19 years [2], pregnant women and individuals with underlying illnesses such as diabetes, asthma, respiratory diseases, immune suppression and renal disease [3]. One dose of pandemic vaccine conferred good protection against the infection in approximately 70% of cases [4]. However, despite the effectiveness of the vaccine, the public demand for vaccination was low and many people were not vaccinated. For example, in the UK uptake of vaccination among clinically at-risk groups was 37.6% [5]. For those who contracted pandemic influenza, antiviral medicines were recommended as a treatment, and the provision of antiviral medicines (also as a preventive measure) was a major component of emergency plans in many countries [6]. Data from the UK National Pandemic Flu Service (NPFS) indicated that of the 1.8 m courses of antiviral medicines that were authorised, only 1.16 million were collected and many patients failed to complete a full course [7]. This suggests that there is a need to develop effective communications to improve uptake and to consider how best to advise the public on the nature of the disease, why they should seek prevention (vaccination) or treatment (antiviral medication), who should seek it and when.

Evidence shows that the factors that have been found to promote uptake of vaccination included being vaccinated for seasonal flu [8-10], perceiving that the outbreak was severe and resulting in high morbidity and mortality [11,12], high levels of worry and anxiety [13], being in a priority group [14] and believing that the vaccine was effective and safe [14,15]. In addition, social influences were important; for example knowing someone who had the disease and knowing that others had a favourable view of the vaccine [11] as well as trust in the source of information [11,15-17]. Factors that have been found to act as barriers to uptake of pandemic influenza vaccination were: believing that the outbreak was not serious [16,17], and not identifying oneself as being at-risk [17]. Fears about the safety and side effects of the vaccine were also a barrier to H1N1 vaccine uptake [8,14,18-20]. It appeared that the public preferred to take the risk of harm posed by the disease over any harm that might be caused by being vaccinated [21,22]. The scant research in the UK and elsewhere about the public’s response to antiviral medicines in the last pandemic suggests that the public knew relatively little about antiviral medicines and had limited experience of their use [23]. Frequent travellers had more positive perceptions of antiviral medication as a result of prior usage [24,25] and research with pregnant women found a tension between women’s desire to protect the foetus from harm and worry about the safety of taking medicines when pregnant [26].

Research conducted with the public in advance of an outbreak can inform the type of messages that are likely to be effective in promoting acceptance of these recommended behaviours [27,28]. Such past research has investigated hypothetical scenarios of varying degrees of severity and advice on a variety of precautionary behaviours including hand-washing, covering the mouth, vaccination and seeking medical attention [29-32]. Results showed that the public was largely unfamiliar with the term ‘pandemic’ and tended to believe that pandemic influenza was similar to seasonal influenza [29,31]. Most people do not know whether the symptoms of pandemic flu are different from pandemic influenza and are unsure how to recognise the signs [29,32,33].

This body of research suggests that, in a future pandemic, the public would benefit from more knowledge about the health threat and about who will be at-risk from infection, how the infection spreads, how to self-diagnose, short and long term consequences of the illness if precautionary measures are not taken, and the potential side effects of vaccination and antiviral drug treatments [30-32,34,35], including safety and efficacy tests for a new vaccine that would be rapidly deployed [30]. In some instances, trust was found to be an important component in acceptance and compliance with recommended behaviours; however, trust in public officials has been found to be weak compared with trust in medical professionals [31,34,36-38]. Although the research described above has identified a range of factors promoting pandemic vaccination, there is less about those factors influencing uptake of antiviral medicines.

While research has often focused on the public’s response to advice during severe or moderate pandemic outbreaks little is known about how the public would respond to advice in an explicitly uncertain situation where the risk is less clear cut. For example, Teasdale & Yardley [32] studied the public’s response to advice in scenarios where the consequences were described as moderate or severe; Elledge and collegues [31] investigated mild and severe scenarios for avian flu and McGlone et al studied [39] responses to a severe scenario. Understanding how the public responds when the progress and impact of a pandemic is uncertain will be important because it is during the emergent, uncertain stages of a pandemic that the public will be asked to consider the potential risk of contracting pandemic influenza and to take precautionary measures to reduce the likelihood of personal infection and spread.

**Figure 1:**
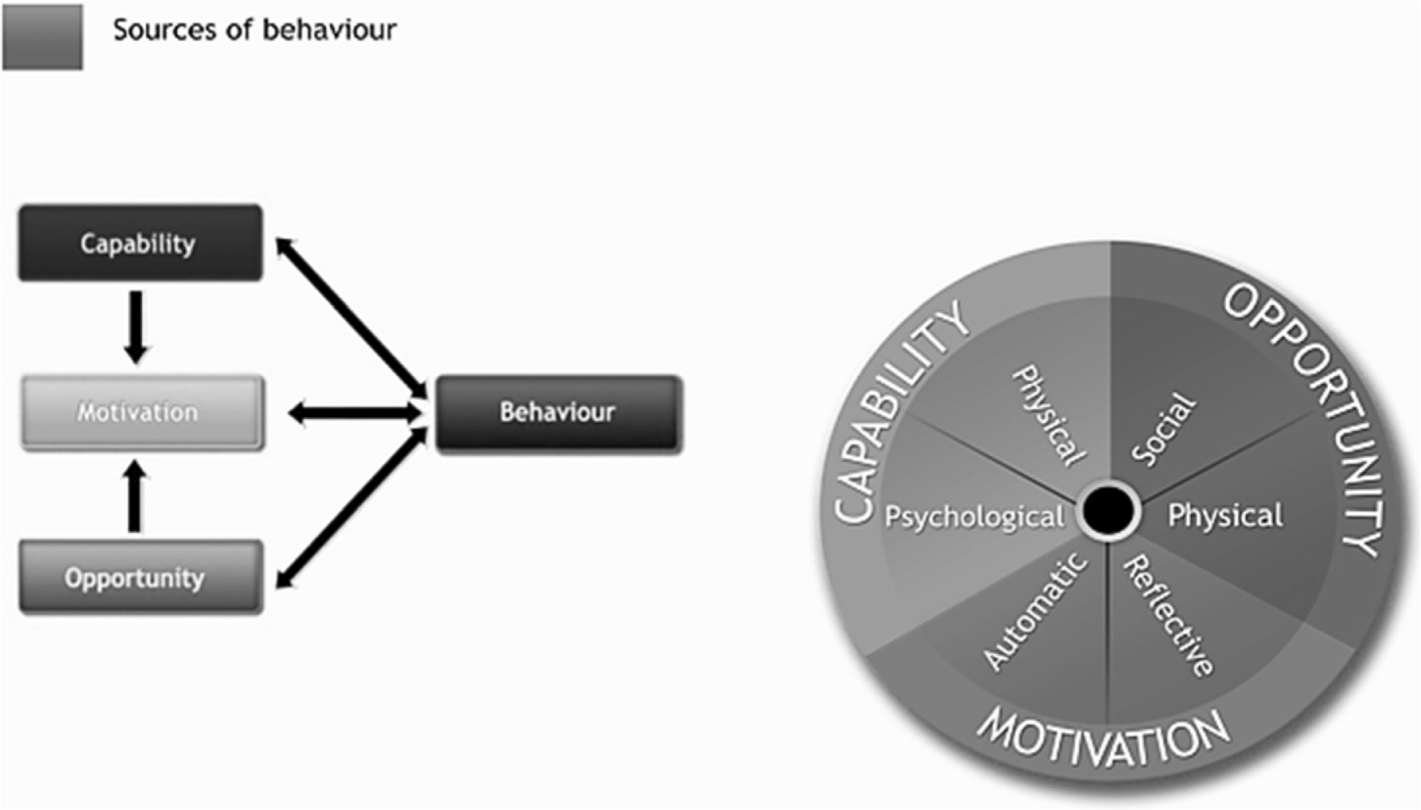
**Schematic of the components and relationships in the COM-B model.**

The majority of studies that have investigated how the public respond to precautionary advice has rarely been informed by a theoretical understanding of behaviour change. Using a theoretical framework helps to integrate empirical findings and elucidate processes of change and mechanisms of action of effective communication and other intervention strategies. A useful framework for this purpose is the COM-B model summarising factors necessary for behaviour to change across behavioural domains [40] (Figure 1). The initials stand for ‘capability’, ‘opportunity’, ‘motivation’ and ‘behaviour’, and the model recognises that behaviour is part of an interacting system involving all these components. Changing behaviour will involve changing one or more of them in such a way as to put the behavioural system into a new configuration and minimise the risk of it reverting. Because of the interacting nature of these components, one may increase, for example, motivation by increasing capability (e.g. knowledge and skills) and opportunity (e.g. access to resources and social influence).

We adopted the COM-B model in our approach to the uptake of pandemic flu vaccination and antiviral medicines because changing the incidence of any behaviour in a group or population is likely to involve changing more than one driver of behaviour.

By specifying the factors that need to change for a behaviour to occur, the model can identify the kinds of interventions that are likely to be effective. The model postulates that for any behaviour to occur a person must have the psychological and physical capability to perform the behaviour; the physical and social opportunity to engage in it, and must be motivated to do so at the relevant moment compared with some other behaviour. Psychological and physical capability refers to the range of capacities such as knowledge, physical and mental skills and facilities such as strength and stamina. Opportunity can be physical and social and refers to environmental factors that permit the behaviour including access, availability, time and financial resources and social factors such as the cultural milieu we operate in. Motivation reflects the brain processes that direct behaviour which may be reflective (evaluations and plans) or automatic (emotions and impulses arising from associated learning). COM-B has been elaborated into 14 theoretical domains, the Theoretical Domains Framework (TDF) [41].

The study aimed to systematically identify facilitators of and barriers to being vaccinated and taking antiviral medicines in uncertain and severe pandemic influenza scenarios using the COM-B framework. An uncertain scenario was used in addition to a severe scenario because in the early stages of a pandemic there is often uncertainty about how the situation will unfold, how rapidly the infection will spread, or what impact this could have on the population. Hence it is important to understand how people respond to precautionary advice in these conditions of uncertainty, how they make sense of the risk, and what types of precautionary measures they express preference for.

## Method

### Design and recruitment

Semi-structured focus groups and interviews were conducted with a diverse sample of the general public. To ensure that participants were from a range of social and ethnic backgrounds we recruited from a variety of organizations in London and Southampton including children’s centres, AgeUK lunch clubs, community centres, students from a university, voluntary organisations and support groups for those with underlying conditions such as diabetes, COPD (Chronic Obstructive Pulmonary Disease) and PSC (Primary Sclerosing Cholingitis). Advertisements were placed in these centres explaining the purpose of the study, who was eligible, how to participate and offering a small monetary compensation for participation. The managers of the centres where interviews were held advertised the study and made rooms available for the focus groups to take place.

Ethical approval for the study was granted by University College London (Reference: 5081/001) and the University of Southampton (Reference: 7387) ethics committees.

### Sample

Sampling was purposeful and individuals who varied in their risk status were recruited. Of the 71 participants, 23 were men and 48 were women; Details of the demographic profile are shown in Table 1. Thirty-five were from designated at-risk groups of whom 10 had an underlying condition, and six were pregnant. Of the 36 participants not designated as being at-risk, nine were specifically recruited because they were mothers with young children. Thirty-eight of the participants were vaccinated for seasonal influenza regularly (of whom 20 were from clinical at-risk groups) and two had been vaccinated for seasonal influenza for the first time this year. Eighteen people who did not consider themselves to be at risk had been vaccinated at least once before for seasonal influenza. Reasons for being vaccinated among those who were designated as not being at high risk included recommendation by a GP, and being offered the vaccine at work. 12 participants had received monovalent H1N1 vaccine and three had antiviral medicines during the 2009-2010 pandemic.

**Table 1:** **Profile characteristics of participants**

*Gender*		*Type of group*	
Male	23	Mothers/young children	9
Female	48	Elderly - aged 65 years and over	19
		Underlying illness*	10
		Pregnant	6
		General public	27
*Location*		*Been vaccinated for seasonal flu*	
London	47	Yes	40
Southampton	24	No	31
*Age*		*Perceived risk of catching influenza*	
16-35 years	21	Not at risk	35
36-64 years	20	At risk	17
65 years and over	30	Not stated	19
*Ethnicity*		*H1N1 vaccine*	
White/White British	42	Yes	12
Black/Black African	22	No	56
Other	7	NA	3
*Education*		*Antiviral for H1N1*	
Secondary school	7	Yes	3
College	7	No	65
University educated	36	NA	3
Not stated	21		

*i.e., diabetic, asthma, COPD.

It should be noted that groups were not always mutually exclusive. For example, some individuals who had been recruited as ‘elderly’ (over 65 years of age) also reported that they had other underlying conditions that would put them in another at-risk category.

### Materials

Two scenarios were developed: an uncertain and a severe scenario. The severe scenario was based on that used by Teasdale and Yardley (2011) which described a severe level of risk, severe health consequences and the national impact of the pandemic. The uncertain scenario was developed to reflect the early conditions that occurred during the 2009/10 pandemic. This described an uncertain situation, uncertain health consequences and uncertain public impact of the pandemic (see Table 2).

**Table 2:** **Uncertain and Severe Scenarios of Pandemic Influenza used in the research**

**Uncertain Scenario**	**Severe scenario**
The [pandemic] flu virus has not yet reached the area where you live but it is now spreading to the UK. In other countries hundreds of people are infected - some people do not have any symptoms but 15 have died.	Flu virus has spread to where you live, **1 in 2 of those coming into close contact with an infected person catch flu.**
**Scientists** do no yet know how badly the flu virus will affect people in the UK **- doctors are trying to learn about the virus as fast as they can, but do not know if it will be mild or serious.**	Most people who catch flu **feel very ill for around a week**. Almost 1 in every 10 people who catch flu need hospital care, and **1 in every 50 healthy people who catch flu die.**
**When the virus reaches the UK,** we don’t know whether life will carry on much as usual or whether there will be serious problems **with services such as the NHS, schools or vital supplies.**	**Life cannot continue as usual.** Most schools close, there is very high sickness absence at work and so there are problems with essential supplies, and health care services are not coping and have to be prioritised for the most seriously ill.

Short messages promoting the uptake of vaccinations and antiviral medicines for pandemic influenza were developed to reflect evidence from prior research that identified barriers to uptake but also to reflect the key drivers of behaviour as defined in the COM-B framework. These were presented as advice from official sources (see Table 3).

**Table 3:** **Advice to take antiviral medicines and to be vaccinated used in the research**

**Antiviral medicines**	**Vaccinations**
PEOPLE WITH PANDEMIC FLU are advised to take antiviral medicines to reduce their symptoms, and the length of time they are ill.	You are advised by your GP to get vaccinated at once to protect you and your family from getting pandemic flu.
PEOPLE IN A PRIORITY GROUP will be provided with antiviral medicines to prevent them from catching flu.	Vaccines for pandemic flu have been through the same careful tests as vaccines for seasonal flu and are safe to use

### Procedure

Data collection took place in London and Southampton from November 2013 to March 2014. Nine focus groups, three paired interviews and six individual interviews were conducted by the first two authors at the centres from which participants were recruited. Written informed consent was obtained from all participants who received a small monetary compensation for their involvement. Interviews lasted between 20 and 65 minutes and were audio recorded with the participants’ consent.

An interview schedule structured into two sections was used to guide the discussion. The first section was to establish what participants knew about pandemic influenza, vaccinations, and antiviral medicines for pandemic influenza, and personal experiences of pandemic influenza. The second section focused on responses to two scenarios and advice concerning vaccinations and antiviral medicines. Participants were asked to imagine that they were in a given situation and to consider what they would think, feel and do if this were to occur. The Uncertain scenario (Table 2) was always shown first, followed by the advice about antiviral medicines (Table 3). The Severe scenario (Table 2) was shown second followed by the advice on vaccinations (Table 3), and then antiviral medicines. All participants were debriefed in full at the end of the interview and reassured that these were fictional scenarios.

### Data analysis

Audio recordings were transcribed verbatim and NVivo 10 was used to code and to maintain a trail of memo and theme development. Analysis was iterative and each transcript was read and re-read numerous times by the first two authors independently. Transcripts were coded line by line and analysed comparatively to identify similarities and differences [42]. A data audit was conducted by the first two authors to clarify meanings, remove duplicated codes and identify data that did not match the coding scheme [43].

Inductive analysis was used to identify responses to the uncertain and severe scenarios. Deductive analysis was used to identify facilitators and barriers to following recommended advice to be vaccinated and take antiviral medicines. In addition, code names were assigned to the six COM-B components: physical and psychological capabilities; automatic and reflective motivations, and social and physical opportunities (see Additional files 1 and 2 – code frames). For the purposes of analysis, the Theoretical Domains Framework [41] was used. This is a variant of the COM-B which subdivides the themes into 14 detailed components that map directly onto COM-B. These are: ‘knowledge’; ‘skills’; ‘memory, attention and decision processes’; ‘behavioural regulation’; ‘social/professional role and identity’; ‘beliefs about capabilities’; ‘optimism’; ‘beliefs about consequences’; ‘intentions’; ‘goals’; ‘reinforcement ‘emotion’; ‘environmental context and resources’; and ‘social influences’^a^.

The facilitators and barriers to being vaccinated and take antiviral medicines were reviewed separately. Responses to accepting advice were also investigated according to two broad categories – those designated as being in a priority group (35 people – men and women over 65 years, pregnant, underlying illnesses) and those not designated as being in a priority group (36 people – men and women under 65 years, mothers with young children).

## Results

Responses to the uncertain and severe scenarios differed: in the uncertain scenario participants were hesitant and ambivalent about following advice because the risk was unclear whereas in the severe scenario the need to act seemed more obvious and almost all claimed they would comply with the official advice.

The focus of this paper is on facilitators and barriers to uptake of pandemic influenza vaccination, because participants knew relatively little about antiviral medicines and were less able to discuss them. Responses to advice about antiviral medicine were more limited, as the participants were largely unfamiliar with these medicines, but were broadly similar to responses to advice about being vaccinated; any differences are highlighted after the responses in common are presented.

### Responses to the scenarios: procrastination vs. call to action

The most common response to the uncertain scenario was to ‘wait and see’ or ‘do nothing yet’. There were two reasons given for this: the situation was likened to the swine flu outbreak, which was not considered to be serious, and it was thought to be distant - both emotionally and physically - and hence, less worrying:

*It hasn’t got into the country at the moment, so um I’m not sure if there are people that have the pandemic flu*. (Pregnant woman^b^)

Personal risk was perceived to be low, even amongst those in a designated priority group. Although there was some evidence to suggest that the uncertainty was experienced as disconcerting, the majority did not see the need for vaccination or antiviral medicines. Rather, participants suggested that they would do more of the behaviours they already practiced such as following good hand and respiratory hygiene and taking more Vitamin C:

*You’d step up your vitamin C etc. and your cod liver oil.* (Male, over 65 years)

*I would be watching more people touching- for me personally, washing my hands er you know being aware if someone sneezes I’d probably ask them to cover their face*. (Mother with young children)

In this situation, it was thought to be important to ‘keep an eye on the media’ to find out what general advice was being given.

In contrast, the most common response to the severe scenario was to take action.

*32 million people in the UK with flu, yeah. Okay. I’m off to the doctor.* (Male, not at-risk)

*You’d probably be ringing up your GP and going ‘I need to look after my daughter - I don’t want to get it. Can you put me in a priority group?* (Female, not at-risk)

The ‘call to action’ occurred because this situation was thought to be serious. ‘Serious’ was often interpreted in terms of the disease being emotionally and physically close rather than in terms of the absolute number of people who were ill, hospitalised or had died.

*If it is your neighbour - it is really – being really ill with flu, if they got it and their baby got it it’s near to you and you know people and I would feel influenced I think. Well I’ve got a baby at home and my elderly mum lives next door I should get it because I don’t want to put them at-risk by me getting or vice versa but if it is on the news and they are telling you in China – you know whatever I am thinking ‘whatever, am I at-risk? Is my family at-risk? It’s on the TV. I don’t know –am I going to get this?* (Female, not at-risk)

In severe scenarios, there was a high level of anxiety and an awareness of personal susceptibility. As one woman with young children commented *‘this is normal people and they are dying’*. The need to take novel precautionary measures, of any kind, was less likely to be disputed:

*I think people follow any advice [in this scenario] that is given from an authority figure anyway, even if it was poison…*(Female, not at–risk)

### Barriers and facilitators to vaccination uptake

Five of the six components in the COM-B model accounted for participants’ responses (Table 4).

**Table 4:** **Factors that can influence uptake of vaccine for pandemic influenza identified in the study using COM-B**

**CAPABILITY**	**MOTIVATION**	**OPPORTUNITY**
*The capacity to engage in the behaviour*	*Brain processes that energise and direct behaviour*	*Factors lying outside the individual that act as barriers or promoters of behaviour*
**Psychological**	**Automatic**	**Physical**
*Capacity to engage in necessary thought processes*	*Emotions and impulses*	*Physical opportunity in the environment*
Knowledge of the disease	Emotion: Fear	Access
• Pandemic influenza is a novel strain	• Expressed not just numerically but in terms of physical and emotional proximity	• to treatments
• Awareness of morbidity, mortality and transmission rates		• to professional advice
Memory	Habitual behaviour	Able to book to see GP
• Media exaggeration of last pandemic	• Being vaccinated for seasonal influenza and taking medicines in general	Avoiding ‘hubs of infection’
**Physical**	**Reflective**	**Social**
*Capacity to engage in necessary physical processes*	*Evaluation and plans*	*Cultural milieu that affects what we think about things*
• Not salient/not mentioned	Beliefs about consequences	Social influences
	• Pandemic influenza is not more serious than seasonal influenza	• Respected others are being vaccinated
	• The vaccine has not been adequately tested and may be unsafe or ineffective	• Believing that it is unacceptable to put others at risk
	Omission bias	Trust
	• Believing that the risks of being vaccinated outweigh the risk of being ill with pandemic influenza	• Recommendation from trusted health professional
		• Respected others recommend
	Identity (health)	Group identity
	• Believing that a healthy lifestyle confers immunity	• Being part of an at-risk support group
	Optimistic bias	
	• Tending to the view that they will not be infected or will make an easy recovery from pandemic influenza	
	Social role	
	• Responsibility for other family members, including unborn	
	Anticipated regret	
	• Concern that the outbreak could be more serious than expected and have not been vaccinated	

### Capability

#### Knowledge

The majority of participants knew little about pandemic influenza and many were unsure of the meaning of the word ‘pandemic’. Overall, few people linked ‘pandemic influenza’ to the A/H1N1 pandemic influenza outbreak of 2009-2010. They tried to make sense of it by likening it to other more familiar phrases such as ‘epidemic’, inferring that it was probably a more widespread and more serious form of influenza:

*I just thought pandemic flu was all kinds of flu, I didn’t…oh well I actually thought maybe pandemic sounds like a flu that is outbreaking and very dangerous and they want to keep it under control*. (Pregnant woman)

Only two people in the study spontaneously referred to the fact that pandemic influenza is a novel strain of virus. When this information was presented, people found the notion of it being a novel strain helpful in explaining the threat it posed beyond seasonal influenza:

*It’s just the word they use when it is worldwide and it is spreading from chickens in China or something, but other than that I didn’t know what it meant, that it was new, why don’t they just say new? I mean they want new, it’s the new one for which there isn’t any vaccine yet; that should be said.* (Female, over 65 years)

In the absence of this new information, some thought that pandemic influenza could be like seasonal influenza.

*What are the symptoms? Are there different symptoms from swine flu and ordinary flu? What would you look out for? How would you know you had one from the other? They could be the same.* (Male, not at-risk)

#### Memory

Some participants spontaneously linked the word ‘pandemic’ to bird flu or swine flu but many did not. Recall of the swine flu pandemic was low, partly because only four participants in our sample had contracted it, and partly because few knew anyone who had. A prevalent comment was that media had exaggerated the risk of swine flu:

*It’s almost like you get kind of a mixed picture of what it actually is, and then, it will be reported in a way that people will think it’s…that they’re not going to be able to avoid catching it or something, and then like…but then, the next day, it will be like, oh, actually, there’s only one person in Yorkshire that’s got it…* (Female, not at-risk)

### Motivations

#### Automatic motivations

In the uncertain scenario, the participants expressed little concern about the pandemic outbreak. Most participants were not worried and so many could not see a need to be vaccinated or take antiviral medicine:

*…there’s nothing to do yet. I feel like this is worrying about nothing* (Male, not at-risk)

*…it’s a good first step, I guess, you know, to try and get the word out there that this could potentially be a problem, but this wouldn’t be the deciding factor [to be vaccinated]*. (Pregnant female)

Having been offered the seasonal influenza vaccine previously was put forward as a reason for considering pandemic influenza vaccination – *‘it would never stop me because I have been having them [seasonal flu jab] for years and years’* (Female, underlying illness).

#### Reflective motivations

In the uncertain scenario, participants tended to make a *‘risk assessment’* (e.g. male, not at-risk) and ‘*weigh up the risk in my mind, the side effects of the vaccine versus am I going to lose my life or be significantly impacted by it’* (e.g. female, underlying condition). Participants deliberated about the consequences of being ill with influenza as opposed to the consequences of being ill with side-effects from the vaccine. In doing so, they drew on their current status as a healthy person who would not need to be vaccinated; on their role in society as a responsible person who should be vaccinated to prevent family members (especially children) from becoming ill; and on feelings of anticipated regret if the virus became worse and they had failed to be vaccinated.

#### Beliefs about consequences

Participants tended to believe that pandemic influenza was similar to seasonal influenza, which was not considered to be a serious illness. If participants thought that the consequences of being ill with pandemic influenza were minimal there was little incentive to take precautionary measures.

*A week being ill [with flu] isn’t the end of the world. I think if I thought it was going to be much worse than that, you know, I would be more concerned and more likely to have the vaccine.* (Female, underlying illness)

There was a view that the consequences of being vaccinated were potentially worse than becoming ill from influenza. In many cases this was related to concerns about side effects or a belief that it was possible to contract influenza from the vaccine itself. These views were not shaped by personal experience.

*What I feel about vaccines is that you actually get a virus or not – what you get is a small amount so you are not supposed to get an illness. I am not sure that is true. I have heard that many people do get ill after having the vaccine…* (Mother with young children)

Only a minority of participants were openly critical of vaccine safety or efficacy but where such concerns were expressed they were given as reasons not to be vaccinated. In expressing scepticism about the safety of a newly developed vaccine the participants drew on beliefs or representations of how drugs are developed and made available to the public, and argued that a pandemic flu vaccine cannot meet the standard safety criteria due to its ‘sudden’ production:

*Every other drug has been tested for years and years before it can go on the shelf. How can they suddenly produce something in six months and put it on the shelf? I’d be very suspicious of that.* (male, not at-risk)

By contrast, a facilitator of vaccine uptake was the belief that a vaccine would be protective. This was of particular relevance to those who were aware that they could have complications as a result of becoming ill, for example, pregnant women who were concerned to protect their babies: *‘It’s only because I’m pregnant that I’m more worried, because otherwise I wouldn’t [be]’.*

A further facilitator of vaccine uptake was anticipated regret: a tendency to consider that the situation could become worse and that there could be negative consequences from not being vaccinated early enough. As this young man who was not at-risk said: *‘It would be a brave man to say no, I’m not taking anything at all when everyone around you is dropping’.*

#### Social identity

Those who were accepting of vaccination and antiviral medicines tended to view themselves as less healthy and acknowledged that they could be at risk of infection from pandemic influenza. They were frequently in contact with medical professionals and followed their advice and routinely took medication and the seasonal influenza vaccination. Many were from a seasonal influenza priority group and regarded the decision to get vaccinated or take medicines as ‘normal’:

*I think if you are already in a group such as us, who are already taking loads of medications, constant checks and tests, you tend to be a bit more accepting. Whereas if you don’t take medications, you’re normally quite healthy and you are suddenly being told ‘we want you to have this, we recommend you take it’.* (Female, underlying illness)

Pregnant women considered themselves to be temporarily in the at-risk category, although most commented that they would prefer not to take medicines in case of harm to the foetus but would do so if a medical professional recommended it.

By comparison, those participants who were less accepting of vaccination advice tended to perceive themselves as ‘fit and healthy’ and have had less frequent contact with medical professionals. Notions of being ‘fit and healthy’, rarely becoming ill and having a strong immune system were invoked to deny the need for vaccination because they were unlikely to be at–risk. A range of behaviours such as, eating healthily and exercising were believed to confer this immunity.

*…look after yourself, eat healthier and do a bit of exercise and try and keep away from people with viruses and that sort of thing and um I do that without sort of getting neurotic about it.* (Male, underlying illness)

Three types of behaviours were commonly cited as a way to stave off infection: social distancing, lifestyle related activities, and improving basic hygiene. More than half of participants spontaneously mentioned distancing behaviours as a means to reduce the risk of being infected, e.g. avoiding crowds, not travelling on public transport, and staying at home:

*I think people will stay indoors, and people will not congregate - meetings or anything like that, supermarkets, trains…* (Male, not at risk)

About one third cited lifestyle behaviours as a means of staving off infection such as eating properly, drinking more water, exercising and supplementing their diet with vitamin C, cod liver oil or orange juice. Finally, improved hygiene behaviour was often mentioned such as using hand gels, washing hands more frequently, cleaning surfaces and covering one’s face when sneezing or coughing.

Using alternative behaviours to vaccination related to the view that medicine should be avoided where possible and that it was better to allow the body to fight off diseases ‘naturally’. Arguably, some people preferred these precautionary behaviours to vaccination because they seemed without side-effects and also more within their direct control. People who held these views could be from either an at-risk or not-at-risk group:

*I would be very happy for my own body to make an attempt to try and fight it because what I know about vaccines is that they break the immune system.* (Female, mother with young children)

*I’m not a great fan of taking medicine for medicines sake really. I think that’s probably the criteria that I applied and I’m just reluctant I think to take something which at the end of the day um I don’t really see the benefit of really*. (Male, not at-risk)

Beliefs about being fit and healthy and being able to naturally fight disease contributed to a sense of optimism: the belief that that they were less vulnerable than others to being infected with pandemic influenza:

*…touch wood, I feel I’m quite healthy anyway…I seem to be alright.* (Pregnant woman)

*I’m alright, I’ll do the best I can, I’ll do my exercise which is my overall shield, my barrier against all diseases…* (Male, underlying illness)

#### Social role

Pregnant women were aware of their social role to protect their unborn child but others also commented that their social role as a protector of their family or as a role model to family members would influence them in the direction of being vaccinated:

*If you are a family person and you have got children that are under sixteen, for example, it’s up to you to decide whether they would have this vaccination, and if you say no, I’m not going to let them have it and they die, that’s a big responsibility on you*. (Male, not at-risk)

*…this is a collective thing* (Female, not at-risk)

*…it’s not just about you is it, it’s about everyone else* (Pregnant woman)

However, only a minority of participants believed that they had a social responsibility to be vaccinated in order to prevent the circulation of the virus within the wider society. Virtually no participant referred to the notion of herd immunity and to the duty of every citizen to vaccinate to reduce others’ risk of infection. Thus, it could be argued that the risk of pandemic influenza was primarily understood as a personal rather social issue, with little attention being paid to the social aspects of a pandemic outbreak.

### Opportunities

#### Physical opportunities

The main physical opportunity that appeared to promote uptake of vaccination was access to advice and treatment. Participants anticipated that vaccination would be readily available at GP surgeries or at pharmacies. However, surgeries were considered to be a ‘hub for infection’ which should be avoided:

*You are going into an environment where you are prone to get flu because there is different people, so I’d be scared. I think I’d be like can’t you just post it through the door, like send it, I don’t know, I wouldn’t go to the centre. Would you?* (Mother with young children)

The anxiety about attending a surgery prompted one participant to suggest that mobile dispensaries should come to local neighbourhoods *‘to bring the medication to you’* (Male, not at-risk). In addition, there was concern about the difficulty of booking an appointment in a timely fashion because of pressures on the health service.

#### Social opportunities

Social influences included recommendations from trusted sources, especially health professionals, taking account of the behaviour of respected others, and the influence of the media.

Participants believed that they would actively seek advice from their GP in a pandemic situation and would put faith in the recommendations made by them because *‘I am not a medic and therefore I follow his advice’* (Male, not at-risk). However, in an uncertain scenario some participants commented that they would seek additional supporting evidence on the internet. Nevertheless, if a GP made a strong recommendation to be vaccinated, most participants would follow their advice:

*If it is very, very strongly recommended [in uncertain scenario], well then I would go and beat the surgery door down and get a vaccine, but um if the advice isn’t that strong well then I’d leave it for a bit and see how I get on.* (Male, underlying illness)

Participants were also likely to respond to sources of informal advice, for example close friends and family, an authority in the workplace or a local community leader. This was particularly evident among a group of elderly Somali women and a group of men in a close-knit area of Central London who said that they would actively seek the advice of community leaders.

Participants acknowledged that the media will play a role during a pandemic outbreak and they expected that they would get information *‘from reliable newspapers not the Sun or Metro’* (Pregnant woman). A common expectation was that the media would exaggerate the situation because ‘*you hear it on the news and you obviously have to take it with a pinch of salt because the news media are always out for a story’* (Male, not at-risk).

#### Group identity

Identifying as being part of a group was a factor in decision-making about vaccination. This was because several people with underlying conditions belonged to support groups either in person or on-line. These groups would sometimes discuss the need for vaccination

*…the people in the online forum talk about flu vaccination…. I know from reading online that it covers people like me* (Female, underlying illness)

However, despite being aware that one was part of an at-risk group, some people who were in the at-risk groups distanced themselves emotionally from the need to be vaccinated. One female participant who had Primary Sclerosing Cholangitis^c^ argued that she would only think of herself as being vulnerable if the people who were infected were from the same country and demographic as herself:

*I think does the risk of getting the vaccine outweigh the risk of the impact on my life. I guess when it is a million miles away and very few people are getting it and it’s a different age demographic to me, I probably think actually I am not going to take that risk [of being vaccinated]…..* (Female, underlying illness)

### Additional factors that may influence uptake of antiviral medicines

Beliefs about antiviral medicines tended to be ill-informed, for example, considering that they were antibiotics and that they would be delivered in injection form.

Many were unsure whether they would recognise the signs of pandemic influenza, for example, ‘What *are the symptoms? Are there different symptoms from swine flu and ordinary flu? What would you look out for? How would you know you had one from the other?’* (Male, not at-risk)

Most of the participants commented that the advice to take antiviral medicines seemed ‘sensible’ and compared with vaccination fewer concerns were raised. Overall there was less resistance to uptake because ‘*if you were feeling ill and feeling like death, you would take anything’* (Male, not at risk).

## Discussion and conclusions

The aim of this study was to systematically identify facilitators of and barriers to being vaccinated and take antiviral medicines in uncertain and severe pandemic influenza scenarios using the COM-B framework. The influences on vaccination and antiviral uptake were wide-ranging, including various aspects of capability, motivation and social opportunity, with some evidence that addressing one aspect could impact on others in the system. For example, social opportunity in the form of recommendations from respected others influenced reflective motivations in the form of beliefs about vaccine efficacy. This suggests that the influences on vaccine and antiviral uptake are multi-dimensional and that communications to promote uptake are likely to be most effective if they address several components.

Identity as a healthy or at-risk individual influenced whether or not people thought they were vulnerable to contracting pandemic influenza and whether they believed that practicing alternative protective behaviours could be as effective as vaccination. Feelings of vulnerability were engendered by being labelled as being in a clinically at-risk group (having an underlying illness, being older or pregnant), and by the severity of the scenario because if it was perceived to be very severe all people will be susceptible to pandemic influenza.

In contrast, those who felt invulnerable to pandemic influenza cited the rarity of being ill with flu and believed that they were, young, healthy or fit and hence had a strong immune system. Those who had constructed an identity as ‘a healthy person’ were less willing to follow advice to be vaccinated and did not view using biomedicine as ‘normalised’. The use of alternative behaviours, especially eating well, exercising and using vitamin supplements was thought to boost immunity and hence, reduce the risk of being infected and the need for vaccination.

Beliefs about being able to boost one’s natural immunity were held by those who were clinically at-risk, as well as by those who were not at-risk. This study suggests that many people in priority groups do not self-identify as being vulnerable and may, therefore, not make the connection with messages aimed at them. Such a disconnection could explain why only 37.65% of those in priority groups in the UK were vaccinated during the last pandemic [5]. More may need to be done to ensure that those in a priority group are able to identify themselves as being more susceptible to the effects of pandemic influenza than others.

Promoters of and barriers to uptake cannot be considered separately from the context of the scenario: in a high risk scenario intentions to follow advice to be vaccinated or to take antiviral medicines were high whereas in the uncertain scenario there was hesitancy and ambivalence and it was in this situation that the full range of doubts, concerns and misperceptions emerged.

COM-B as a framework for analysis was a useful starting point for identifying the range of factors associated with uptake of vaccination and antiviral medicines. The barriers and facilitators of uptake could be classified within the framework which allowed an explanation of behaviour across several components. Many of the factors discussed have been identified in previous studies; for example this study supports previous research that one of the most consistent predictors of vaccine uptake is the habit of being vaccinated for seasonal influenza [8-10,14,19], that the role of emotion (automatic motivations) is highly relevant [11] and that a barrier to vaccine uptake is negative beliefs about the vaccine such that the consequences of being vaccinated are perceived to be as or more problematic than the consequences of becoming ill with pandemic influenza [18,19,21,33,44,45].

However, comparison between studies is made difficult because different researchers select a small sub-set of predictor variables to examine; only a minority make use of a model of behaviour to explain why these variables were selected (exceptions are Teasdale & Yardley 2011 [27], Myers & Goodwin 2012 [46], and Kok et al 2010 [12]) or accommodate different levels of severity.

COM-B is a theoretical starting point for understanding behaviour within specific contexts and to make a ‘behavioural diagnosis’ of what needs to change to alter behaviour. It is at the centre of the Behaviour Change Wheel [40] - a tool to guide intervention design by identifying which intervention functions are likely to be most effective. It is beyond the scope of this paper to enumerate the range of potential interventions but a few examples are described below:

- The study indicated that identity as a ‘healthy person’ was a barrier to being vaccinated. Messages that address these beliefs – for example, explaining how no-one is immune to a new strain of flu and that being vaccinated can enhance health by boosting immunity – may be effective in increasing uptake.
- A further barrier to uptake was a belief that lifestyle behaviours such as eating healthily and exercising could confer immunity and make people less vulnerable to contracting pandemic influenza. Communications that address these beliefs might include information about why people are vulnerable to a new strain of influenza and about the effectiveness of vaccines in reducing the risk of infection or in boosting immunity.

Although the participants were purposively sampled to represent a range of risk profiles, a limitation of this research was that the sample may not reflect the views of the wider population because it was not representative and focus groups may attract people who are particularly interested in the topic area. Furthermore, it is not clear whether being in the habit of being vaccinated or not being vaccinated conditioned responses to different scenarios. This could be explored in future research.

Future research needs to take account of the extent to which messages about vaccination can be transparent in addressing concerns about the vaccine; for example being more open about how the vaccine is developed. In addition, we should investigate whether messages that address identity are effective in promoting uptake of vaccination. In particular, to examine whether positively framed health messages that focus on wellbeing are more effective than messages about risk reduction for individuals who do not self-identify as being vulnerable to infection.

The promoters and barriers to being vaccinated and taking antiviral medicines are multi-dimensional, and communications to promote uptake are likely to be most effective if they address several components of behaviour. The benefit of using the COM-B model is that it is at the core of an approach that can identify effective strategies for behaviour change or communications for the future. People from at-risk groups do not always perceive themselves to be at-risk because they have constructed an identity as a healthy person who is immune from infection because they follow a healthy lifestyle. Communications should confront these identity beliefs by addressing how vaccination can boost wellbeing and immunity.

## Endnotes

^a^The original TDF was developed by an international panel of 32 experts in behaviour change who identified 128 constructs from 33 behaviour change theories and simplified them into domains. Usability was developed with an international team of implementation scientists. The TDF has been validated and refined by an international panel of 36 experts in behaviour change.

^b^Participants are referred to by gender and whether they are in an at-risk group (over 65 years, pregnant, underlying illness) or not in an at-risk group (included mothers with young children).

^c^PSC is a disease of the liver and people with this condition are recommended to have the influenza vaccine because they have lowered immunity as a result of the treatments they receive.

## Additional files

Additional file 1:
**Coding frame: responses to uncertain and severe scenario.**
Additional file 2:
**Code frame: barriers and promoters of uptake.**
