# Supplementary material for: Public preferences for vaccination and antiviral medicines under different pandemic flu outbreak scenarios
Source: BMC Public Health. 2015 Feb 27;15:190. doi: 10.1186/s12889-015-1541-8 (PMC4350649; doi:10.1186/s12889-015-1541-8)
Supplement: Additional file 1: — Coding frame: responses to uncertain and severe scenario. [file 12889_2015_1541_MOESM1_ESM.docx]

**ADDITIONAL FILE 1**

**CODING FRAME: RESPONSES TO UNCERTAIN AND SEVERE SCENARIO**

| **Uncertain scenario – overarching theme PROCRASTINATION** | | |
| --- | --- | --- |
| **Code** | **SUB THEME** | **Example quotation (s)** |
| Spontaneous comments  Expressions of concern  Reactive | Indifference | *I would feel like they’re trying to em…they’re preparing for the worst-case scenario, and in that kind of sense, it’s like selling it to me, with all this precautions, or all of these worries and concerns*.  *There’s nothing to do yet. I feel like this is worrying about nothing.*  *I would be alert and keep an eye on the media*  *I wouldn’t be worried at all to be honest*  *Even if that’s true, let’s say a simple personal cost benefit analysis, you know, like there is so many other things you should be worried about.* |
| Perceived severity  Like seasonal flu  Same as swine flu | Distant | *Because it hasn’t got into the country at the moment, so um I’m not sure if there are people that have the pandemic flu.*  *Same as swine flu – you have to go and work*  *Interviewer: So does this seem any different from ordinary flu?*  *Participant: Maybe not. I suppose if there’s a high death rate then it would be maybe worse because people die in cold weather, don’t they.*  *I don’t know anybody who died from that or who’s seriously ill from any of these viruses.* |
|  |  | *Interviewer: So does this seem any different from ordinary flu?*  Participant: Maybe not. I suppose if there’s a high death rate then it would be maybe worse because people die in cold weather, don’t they. |
| Protective measures:  Carry on as usual  Keeping an eye out  Take vitamin C | The same as usual but more so | *When I am out I would be watching more people touching- for me personally, washing my hands er you know being aware if someone sneezes I’d probably ask them to cover their face. You know don’t do it to me now but stop doing you are spreading your germs so for me it is more of a thing of touching things* |
| Old people will get it  Children  Vulnerable people | Low perceptions of risk: others not me | *if those fifteen people were um more elderly or very ill children um I would be probably be less concerned about it*  *it would depend on the categories which those people were*  *All vulnerable people, ain’t it* |
| Read papers  Search internet  Keep an eye out | Information seeking about what to think | *Keep an eye on the media, and um I think we’d just watch...sort of see what the general advice is* |
| **Severe scenario – overarching theme – URGENCY** | | |
| Spontaneous comments  Expression of concern | Call to action | *I would isolate myself*  *I would feel quite anxious*  *N: you’d probably be ringing up your GP and going ‘I need to look after my daughter, I need to look after my daughter. I don’t want to get it. Can you put me in a priority group*  *I’d be frightened man, I would want….I’d lock up my house…* |
| Perceived severity  Proximity | Emotionally and physically close | *I mean, in scenario two, this is happening right now*  *This one [severe scenario] is where I live. One or two people are catching it. And that one’s fifteen people could have died anywhere*  *I’d panic more on number two because if it’s in my home town I’d definitely have to run and go and get checked out.* |
| Protective measures:  Hoarding  Vaccination  Distancing | Distancing and prevention | *Most schools close. I think many people will stay at home, they won’t leave place.*  *I think better to...if you really worried you can’t leave home then better to get advice online, NHS Direct, then what they recommend and pop to GP, I mean pop to pharmacy and get it [vaccine].*  *If you were ill and feeling like death you would do anything* |
| Anyone can get it | High perception of risk | *It is everywhere now*  *This is normal people and they are dying*    *Everyone is ill* |
| Demand for more information  Information from GP  Advice from Chief Medical officer  Where to go | Information seeking about what to do | *I would try and get as much information as possible from reliable sources, like a GP or someone that is a doctor and knows about this specific situation, and then act accordingly.*  *I think people follow any advice that is given, em, from an authority figure anyway, even if it was poison [laughing]. If they were told from an authority figure to take it…* |
