# Supplementary material for: Public preferences for vaccination and antiviral medicines under different pandemic flu outbreak scenarios
Source: BMC Public Health. 2015 Feb 27;15:190. doi: 10.1186/s12889-015-1541-8 (PMC4350649; doi:10.1186/s12889-015-1541-8)
Supplement: Additional file 2: — Code frame: barriers and promoters of uptake. [file 12889_2015_1541_MOESM2_ESM.docx]

**ADDITIONAL FILE 2**

**CODE FRAME: BARRIERS AND PROMOTERS OF UPTAKE**

| **COM-B components – themes identified in the study** | **Relevant constructs from Theoretical Domains Framework that map onto COM-B components** | **Codes mapped onto TDF framework** | **Example quotation (s)** |
| --- | --- | --- | --- |
| **Psychological CAPABILITY –** the range of capacities needed to perform the behaviour | Awareness, knowledge understanding, memory of the   - disease - vaccine - antivirals | Acknowledged ignorance of pandemic flu  Partial knowledge of pandemic flu  Meaning of word ‘pandemic’  Susceptibility to pandemic flu  Understanding of what vaccines do  Knowledge of antiviral medicines  Memory:  Swine flu had little impact/was exaggerated  Personal experience of swine flu  Geographical source of swine flu | Disease: *“What’s the difference between flu and pandemic flu? Is it a big explosion of phenomenal flu?”*  Vaccine: *“They put a little bit of the flu inside you so your body is immune to it, is that right?”*  Antivirals: *“Won’t it just be like antibiotics?”*  Memory: *As I say, I don’t care where they came from but as I understand it, from the newspapers, they’ve come from Asia.* |
| **Physical CAPABILITY** | Skills | NA | Not mentioned |
| **Social OPPORTUNITY –** the social environment that enables or prevents the behaviour from being performed | Social influences | Others views/others recommend  Views of health professionals  Trust in health professionals  Views of community leaders  Media information | *“…and I remember somebody saying, “Oh well, it’s very selfish of you not to have it,” because, you know, then you’re actually putting other people at risk”.*  *“I’m really keen on medicine. If doctor recommends something I have to finish it”.* |
|  | Social identity: Group identity (as being at risk or not at–risk) | Identify with group  Identify with similar people | *“If you are young, strong and fit you are more likely to resist it sort of thing”.*  *“…if you are already in a group such as us who are already taking loads of medications, constant checks and tests, you tend to be a bit more accepting. Whereas if you don’t take medications, you’re normally quite healthy and you are suddenly being told we want you to have this, we recommend you take it; then perhaps you are...”* |
| **Physical OPPORTUNITY –** the physical environment that enables or prevents the behaviour from being performed | Environmental context that encourages or discourages a behaviour  Barriers and facilitators to adaptive behaviour | Avoid hub for infection  Difficulty of getting appointment  Being offered the vaccine/  Encouragement from doctor or nurse to give vaccine  Being prescribed the antiviral | *“You go to any medical centre, and it’s a hub of infection”.*  *“I’d rather them come to my house, at the door they give me the medication, and I’m happy to take it but come to my house”.*  *“Booking an appointment (with GP) can be hard”* |
| **Automatic MOTIVATIONS –** the processes that involve emotional reactions impulses or reflex responses | Emotions such as fear and anxiety | Worry  Fear | *“I’d be sacred to open up my own letterbox because I wouldn’t know what was in there”* (severe scenario)  *“I’m not going to worry about it ”(*uncertain scenario) |
|  | Habitual behaviour | Seasonal flu injection  Taking medicines | *“I already get the flu-jab now, em, and I can remember how unpleasant it is the last time I had the flu or anything related, similar to it, em, I think I would probably go for the preventative as well, in the first place”* |
| **Reflective MOTIVATIONS –** planning and evaluations that are involved in deciding to perform the behaviour | Social role | Protect family  Protect unborn child  Act as role model | *“…on my own I can make my own choice but where I’m pregnant I’m carrying a life, I can’t just choose what I want for my life and not regard this life as well”*  *“If you are a family person and you have got children that are under sixteen, for example, it’s up to you to decide whether they would have this vaccination, and if you say no, I’m not going to let them have it and they die, that’s a big responsibility on you”.* |
|  | Social identity (health related) | Avoid medicines  Allow body to combat illness  Prefer natural remedies  Importance of following doctor’s advice  Not someone who gets ill  Have strong immune system | *“I avoid taking antibiotics as much as I can, and I think I have quite a strong immune system”*  *“I prefer to do it the natural way. And I haven’t…I don’t get sick too easily”.* |
|  | Optimism – the confidence that things will happen for the best | Feel healthy  Exercising and eating well are preventative  Not at risk because strong immune system  Fatalism | *“I’ve always had that set in my mind that, you know, it’s like…yeah… You get sick, you get better [laughing], you know what I mean?!”* |
|  | Beliefs about capabilities- self efficacy | Ease of following advice  Ability to self-diagnose | *“In my opinion, (it) will be easy to follow (advice). On the other hand, it won’t be easy with kids to follow”.*  *“I wouldn’t really know if I had swine flu. I mean, I wouldn’t know what to…what the symptoms were and what to look out for. How many people would?!”* |
|  | Beliefs about consequences of the   - disease - vaccine - antivirals | Disease   - Children/elderly affected(but not me) - Can spread quickly - Can mutate - Need to stockpile drugs - Severe scenario   - Would affect me   - High sickness   - Absence at work   - Schools shut   - Burden on health service - Uncertain scenario   - Not affect me   - Just bad flu   Vaccine:   - Will protect/prevent - Not deadly - Given small dose of the disease to build immunity - Can give you flu/ are side effects - Not 100% protective - Suspicious of what injecting into body - Risky for young children - Risky for baby/unborn   Developed quickly so how safe is new vaccine?   - Stop working for new strain - Seasonal jab will be effective   Antivirals:   - Alleviates/reduce symptoms - Builds immunity - Take when already ill - Won’t cure/won’t prevent/not solving problem - Works like antibiotics - Might react to medication/side effects - Good when don’t yet know cause of pandemic | Disease – severe scenario: *“So that would be very significant to me and that would really impact on me personally”.*  Disease: -uncertain scenario: *“There’s nothing to do yet. I feel like this is worrying about nothing”*  Antiviral medicines: *“You won’t feel better but it will reduce your symptoms. So it won’t cure it. This antiviral doesn’t cure it”.*  Vaccine: *“…they’ve all been tested but I’ve never had nothing. No one’s died from having a flu jab”*  Vs.  Vaccine: *“this is a new virus, and therefore it may…the normal testing process may not apply to a new virus”* |
|  | Anticipated regret | Anticipated regret | Vaccine: *“It would be a brave man to say no, I’m not taking anything at all when everyone around you is dropping”* |
|  | Intentions to   - be vaccinated - get antivirals - get information | Intentions to be vaccinated  Intentions to get antiviral medicines  Intentions to get information | *“I want the vaccination but if it is going to take too long while I am waiting I want a prescription for antivirals”*  *“I think it depends on how bad you get it. If I felt I was really ill, I would take (antivirals)”*  *“I would try and get as much information as possible from reliable sources, like a GP or someone that is a doctor and knows about this specific situation, and then act accordingly”.* |
